# Scaling of mortality in 742 metropolitan areas of the Americas

**DOI:** 10.1126/sciadv.abl6325

**Published:** 2021-12-08

**Authors:** Usama Bilal, Caio P. de Castro, Tania Alfaro, Tonatiuh Barrientos-Gutierrez, Mauricio L. Barreto, Carlos M. Leveau, Kevin Martinez-Folgar, J. Jaime Miranda, Felipe Montes, Pricila Mullachery, Maria Fatima Pina, Daniel A. Rodriguez, Gervasio F. dos Santos, Roberto F. S. Andrade, Ana V. Diez Roux

**Affiliations:** 1 Urban Health Collaborative, Drexel Dornsife School of Public Health, Philadelphia, PA, USA.; 2 Department of Epidemiology and Biostatistics, Drexel Dornsife School of Public Health, Philadelphia, PA, USA.; 3 Center for Data and Knowledge Integration for Health (CIDACS), Fiocruz, Salvador, Bahia, Brazil.; 4 Institute of Physics, Federal University of Bahia, Salvador, Bahia, Brazil.; 5 Escuela de Salud Pública, Universidad de Chile, Santiago de Chile, Chile.; 6 Instituto Nacional de Salud Pública, Cuernavaca, Mexico.; 7 Institute of Collective Health, Federal University of Bahia, Salvador, Bahia, Brazil.; 8 Instituto de Producción, Economía y Trabajo, Universidad Nacional de Lanús, Buenos Aires, Argentina.; 9 Consejo Nacional de Investigaciones Científicas y Técnicas, Buenos Aires, Argentina.; 10 CRONICAS Centre of Excellence in Chronic Diseases, Universidad Peruana Cayetano Heredia, Lima, Peru.; 11 School of Medicine, Universidad Peruana Cayetano Heredia, Lima, Peru.; 12 Department of Industrial Engineering, Universidad de los Andes, Bogotá, Colombia.; 13 Institute for Information and Communication on Health—ICICT/FIOCRUZ, Rio de Janeiro, Brazil.; 14 i3S—Instituto de Investigação e Inovação em Saúde, Porto, Portugal.; 15 Department of City and Regional Planning, University of California, Berkeley, Berkeley, CA, USA.; 16 Economics Faculty, Federal University of Bahia, Salvador, Bahia, Brazil.

## Abstract

We explored how mortality scales with city population size using vital registration and population data from 742 cities in 10 Latin American countries and the United States. We found that more populated cities had lower mortality (sublinear scaling), driven by a sublinear pattern in U.S. cities, while Latin American cities had similar mortality across city sizes. Sexually transmitted infections and homicides showed higher rates in larger cities (superlinear scaling). Tuberculosis mortality behaved sublinearly in U.S. and Mexican cities and superlinearly in other Latin American cities. Other communicable, maternal, neonatal, and nutritional deaths, and deaths due to noncommunicable diseases were generally sublinear in the United States and linear or superlinear in Latin America. Our findings reveal distinct patterns across the Americas, suggesting no universal relation between city size and mortality, pointing to the importance of understanding the processes that explain heterogeneity in scaling behavior or mortality to further advance urban health policies.

## Teaser

The scaling of mortality in cities of the Americas varies widely by cause and region.

## INTRODUCTION

More than 50% of the global population lives in cities, and by 2050, this figure is expected to reach 70% (*1*). The process of urbanization has been especially intense in Latin America, a region that has undergone a rapid urbanization process in a brief period of time. North America also exhibits high levels of urbanization, making the Americas the most urbanized region in the world (*2*). Cities are complex systems of interacting agents that give rise to emergent phenomena, including levels and distributions of population health (*3*–*6*). Although much research has evidenced contrasting levels of health between urban and rural areas, findings have been mixed partly due to the heterogeneity of urban areas themselves (*6*). Our knowledge of what specific features of urban areas or cities affect population health, and of the processes through which they do so, is still limited (*7*).

Like biological organisms, as cities grow the complexity of their processes also grows (*4*, *5*, *8*). Population size can be conceptualized as an indicator of multiple socioenvironmental mechanisms linked to the agglomeration and intense interaction of people, which can result in advantages or disadvantages depending on the outcome in question. Some processes scale superlinearly with the population size of cities, including socially generated outcomes such as wealth and crime, meaning that they occur at a higher rate in larger cities, potentially due to network effects and increased social interactions (*4*, *5*). Other processes scale sublinearly, meaning that they increase at a slower rate than the population; for instance, infrastructure characteristics such as the road network length scale sublinearly (*4*, *5*), leveraging economies of scale, such as the ability of roads to accommodate a higher number of users. Last, a number of outputs scale linearly, meaning that they occur at similar rates across the continuum of population size (*4*, *5*). Exploring the specific processes that link health outcomes and population size in cities may shed light into underlying mechanisms that could be harnessed to improve population health in urban areas.

To date, a number of studies have examined how population size relates to health in cities (*4*, *9*–*19*). Here, we report on a comprehensive examination of how mortality scales with city population size across the universe of 742 metropolitan areas of 100,000 residents or more in the United States and 10 Latin American countries. We advance and innovate on previous work in three ways. First, by investigating cities across 11 very different countries, we can draw more generalizable inferences regarding the urban scaling of mortality. Second, we provide an exhaustive examination of both fine-grained and coarse classifications of causes of death. Third, we conduct the analyses taking into consideration key epidemiologic and demographic aspects that may drive differences in mortality linked to city size, including age differentials across cities and coding differences across countries. These results provide a comprehensive understanding of how health indicators scale with city size. Knowledge of the fundamental drivers of health in cities is critical to identifying the strategies that can be used to promote health in urban areas.

## RESULTS

### Urban scaling of all-cause mortality

**Fig. 1.**
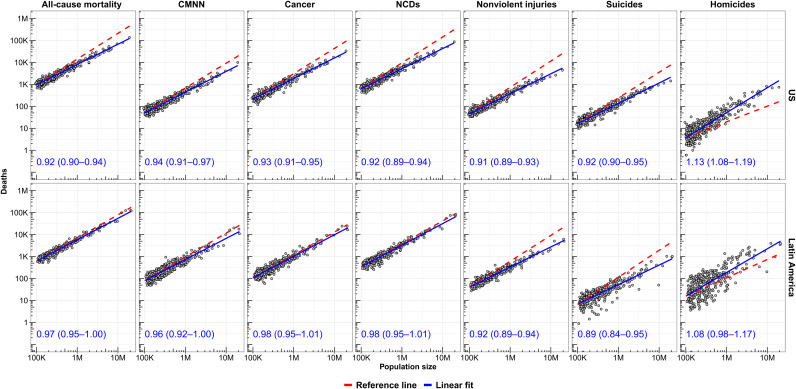
Scaling of all-cause and cause-specific mortality relative to city population size in U.S. and Latin American cities. Solid blue lines are linear fits of log(deaths) on log(population); red dashed lines are reference lines (β = 1). Coefficients (95% CI) are unadjusted coefficients of log(deaths) on log(population), stratified by region. CMNN, communicable, maternal, neonatal, and nutritional conditions; NCDs, noncommunicable diseases.

We first explored the scaling properties of all-cause and cause-specific mortality in 742 cities pooling data from 2010 to 2016. We found that all-cause mortality scaled sublinearly, with β = 0.94 [95% confidence interval (CI), 0.92 to 0.96] (Fig. 1, Table 1, and fig. S1). In other words, more populated cities have a relatively lower mortality rate compared to smaller cities. After adjusting for age and country, the sublinear scaling coefficient was minimally attenuated (β = 0.97; 95% CI, 0.96 to 0.97), indicating that, even after considering the different distribution of ages across cities among the entire region of the Americas, larger cities had lower all-cause mortality. We also found that this pattern differed by region. Specifically, after adjusting for age and country, the scaling coefficient for all-cause mortality was sublinear in U.S. cities (β = 0.94; 95% CI, 0.93 to 0.95) but linear in Latin American cities (β = 1.00; 95% CI, 0.99 to 1.01). Therefore, the health advantage of living in larger cities was present in U.S. cities and was absent in Latin American cities. The scaling pattern for Latin American cities was similar in cities of the two largest countries, Mexico (β = 1.01; 95% CI, 0.98 to 1.03) and Brazil (β = 1.00; 95% CI, 0.99 to 1.01), and other Latin American countries (β = 0.99; 95% CI, 0.97 to 1.01).

**Table 1.** Scaling coefficients (β, 95% CI) by cause of death for all U.S. and Latin American cities. CMNN, communicable, maternal, neonatal, and nutritional diseases; CVD/NCDs, cardiovascular disease and other noncommunicable diseases.

**Cause/group**	**Unadjusted**	Adjusted*	**United States^†^**	**Latin America^†^**	**BR^†^**	**MX^†^**	**Latin America** **(no MX/BR)^†^**
All-cause mortality	0.94 (0.92–0.96)	0.97 (0.96–0.97)	0.94 (0.93–0.95)	1.00 (0.99–1.01)	1.00 (0.99–1.01)	1.01 (0.98–1.03)	0.99 (0.97–1.01)
CMNN	0.96 (0.93–0.99)	0.97 (0.95–0.99)	0.95 (0.92–0.97)	1.01 (0.98–1.03)	1.01 (0.98–1.04)	0.99 (0.95–1.03)	1.02 (0.98–1.07)
Cancer	0.94 (0.91–0.97)	0.98 (0.97–0.99)	0.95 (0.94–0.97)	1.01 (1.00–1.03)	1.01 (1.00–1.03)	1.01 (0.98–1.04)	1.00 (0.97–1.03)
CVD/NCDs	0.93 (0.91–0.96)	0.96 (0.95–0.97)	0.94 (0.92–0.95)	1.00 (0.99–1.01)	0.99 (0.98–1.01)	1.02 (0.99–1.04)	0.99 (0.96–1.01)
Nonviolent injuries	0.91 (0.89–0.93)	0.93 (0.91–0.94)	0.92 (0.90–0.94)	0.93 (0.90–0.95)	0.93 (0.90–0.97)	0.94 (0.90–0.99)	0.90 (0.85–0.95)
Suicides	0.88 (0.84–0.93)	0.92 (0.89–0.94)	0.94 (0.92–0.97)	0.88 (0.84–0.92)	0.88 (0.83–0.93)	0.91 (0.82–1.00)	0.87 (0.79–0.95)
Homicides	1.14 (1.07–1.22)	1.12 (1.07–1.16)	1.12 (1.07–1.18)	1.10 (1.04–1.17)	1.17 (1.09–1.25)	0.97 (0.80–1.13)	1.01 (0.91–1.12)

*Adjusted model is adjusted by age structure and country.

^†^Stratified models are run only on the indicated sample (e.g., Latin America is ran with all Latin American cities), all adjusted by age structure and country (where relevant). For Latin American cities, the main analysis includes the 366 cities in 10 countries, while BR includes Brazilian cities (*n* = 152), MX includes Mexican cities only (*n* = 92), and “no BR/MX” includes all Latin American cities except for those in BR and MX (*n* = 122).

### Urban scaling of large groupings of causes of death

Next, to determine whether these overall scaling patterns of all-cause mortality obscure differing patterns by cause, we explored whether the sublinear (for the United States) and linear (for Latin America) scaling of all-cause mortality applied to the six large groupings of causes of death (Table 1). The three large disease groupings [CMNN (communicable, maternal, neonatal, and nutritional condition), cancer, and NCDs (noncommunicable diseases)] had sublinear scaling in U.S. cities (β = 0.95, 0.95, and 0.94) and linear scaling in Latin American cities (β = 1.00, 1.01, and 1.01). In contrast, nonviolent injuries (β = 0.92 in the United States and 0.93 in Latin America) and suicides (β = 0.94 and 0.88) had a sublinear pattern in both regions, while homicides had a superlinear pattern (β = 1.12 and 1.10). The sublinear pattern for nonviolent injuries and suicides for Latin America was similar for Brazilian, Mexican, and all other Latin American cities (β = 0.93, 0.94, and 0.90 for nonviolent injuries and β = 0.88, 0.91, and 0.87 for suicides). However, the superlinear pattern for homicides varied widely, being superlinear only for Brazilian cities (β = 1.17) and linear for Mexican and other Latin American cities (β = 0.97 and 1.01).

**Fig. 2.**
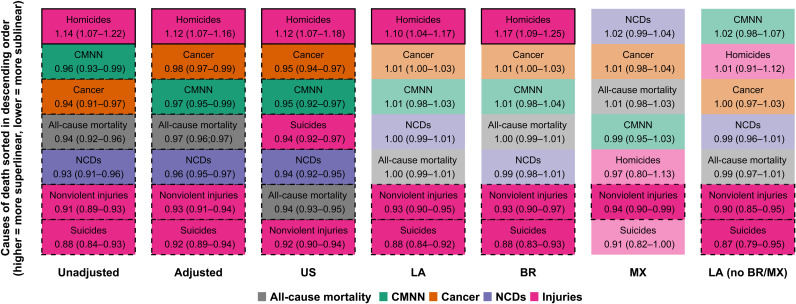
Large groupings of causes of death sorted by scaling coefficient. Fully colored cells indicate a statistically significant superlinear or sublinear pattern; cells with a solid outline indicate a superlinear pattern; cells with a dashed outline indicate a sublinear pattern; non–fully colored cells with no outline indicate a coefficient whose 95% CI crosses the null of linearity.

Of note, model fit for most outcomes by region was very good, with all *R*^2^ above 80% and most above 90%, with the exception of homicides in Mexico (*R*^2^ = 65%, compared to 81% in the United States, 83% for all cities in Latin America, 87% for Brazil, and 89% for all other Latin American cities; see table S2), indicating a higher variability in homicide rates across cities in Mexico after accounting for city size, suggesting stronger place-based (city-specific) effects in Mexico. Later in this section, we will describe a more comprehensive exploration of the relationship between this variability in outcomes and scaling behaviors. Figure 2 summarizes the ordering of scaling coefficients by model, with causes of death that are more superlinear on top. We note that, irrespectively of the superlinear, linear, or sublinear character of the results, the ordering of the six large groupings of causes of death was similar for U.S. and Latin American cities as a whole. This means that, while the specific scaling coefficients varied by region, the ordering of causes from most to least superlinear was mostly conserved. The only exception was suicides, which were the most sublinear cause in Latin America and the fourth most sublinear cause in U.S. cities. Brazilian cities followed a very similar pattern to Latin American cities as a whole. In contrast, in Mexican cities, homicides ranked only fifth in terms of superlinearity (as compared to first in U.S. cities, Latin American cities as a whole, and Brazilian cities). NCDs (which were linear for the Latin American region as a whole) were weakly superlinear in Mexico and ranked first in terms of superlinearity (as compared to fifth, fourth, and fifth in U.S. cities, Latin American cities as a whole, and Brazilian cities, respectively).

### Urban scaling of detailed causes of death

**Fig. 3.**
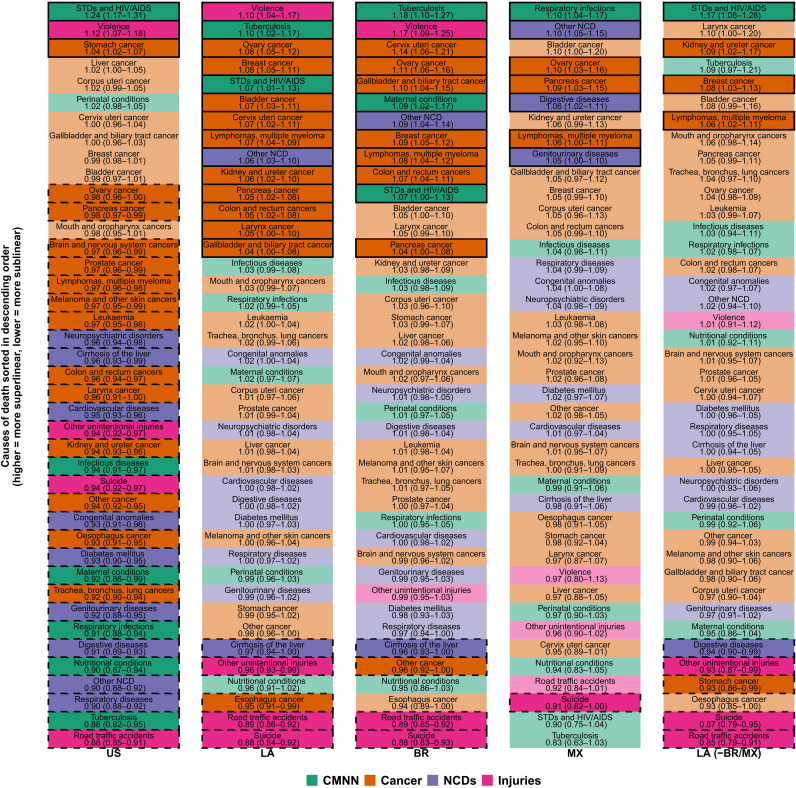
Causes of death sorted by scaling exponent and region. Fully colored cells indicate a statistically significant superlinear or sublinear pattern; cells with a solid outline indicate a superlinear pattern; cells with a dashed outline indicate a sublinear pattern; non–fully colored cells with no outline indicate a coefficient whose 95% CI crosses the null of linearity.

**Fig. 4.**
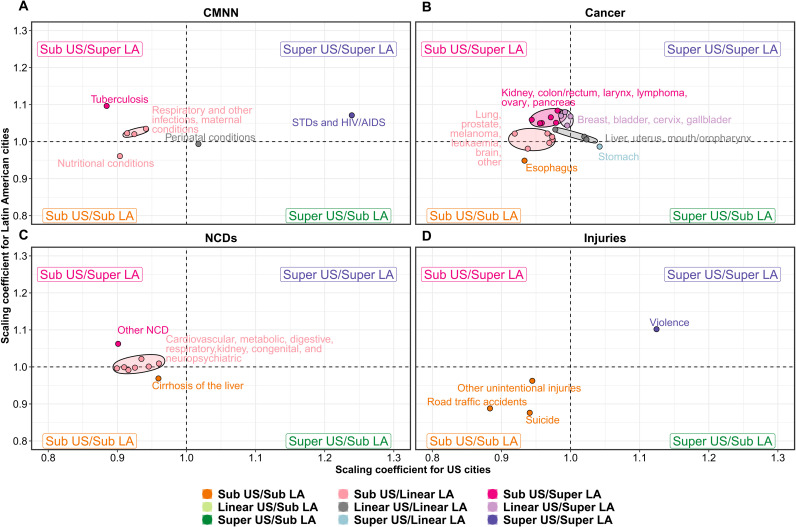
Comparison of scaling coefficients in U.S. versus Latin American cities by cause. Coefficients come from a model adjusted for country and age distribution and stratified by region (Eq. 3).

Given the heterogeneity in causes of death within these large six groupings, we also conducted a more fine-grained analysis focused on specific death causes (see Figs. 3 and 4 and tables S3 to S5 for results).

### Communicable, maternal, neonatal, and nutritional conditions

Among the four communicable disease conditions [sexually transmitted diseases (STDs) and HIV/AIDS, tuberculosis, respiratory infections, and all other infectious diseases], we found different patterns by region and cause. STDs and HIV/AIDS had a superlinear behavior for U.S. cities, Latin American cities as a whole, Brazilian cities, and all other Latin American cities (β = 1.24, 1.07, 1.07, and 1.17, respectively), but they were sublinear in Mexico (β = 0.90). STDs and HIV/AIDS were actually the most superlinear cause of death in U.S. cities and in Latin American cities excluding Brazil and Mexico, but they were the second most sublinear cause in Mexico.

On the other hand, tuberculosis had a very different pattern in U.S. and Latin American cities (Fig. 4A): It was the second most superlinear cause in Latin American cities but the second most sublinear cause in U.S. cities (β = 1.10 and 0.88 for Latin American and U.S. cities, respectively). The pattern in Latin American cities was driven by Brazil and all other Latin American cities (β = 1.18 and 1.09, respectively), as tuberculosis was the most sublinear cause in Mexican cities (β = 0.83). In general, all other communicable disease conditions, along with maternal, neonatal, and nutritional conditions, had a sublinear behavior in U.S. cities (ranking among the most sublinear causes of death) and a linear behavior in Latin American cities, with two exceptions: Maternal conditions were superlinear (β = 1.09) in Brazilian cities, and respiratory infections were superlinear in Mexican cities (β = 1.10).

### Noncommunicable diseases

We observed that mortality from most cancers showed a sublinear (13 of the 21 subcategories) or linear (7 of 21) pattern in U.S. cities. In contrast, cancer deaths showed a linear (10 of 21) or superlinear (10 of 21) pattern in Latin American cities. Specifically, and as seen in Fig. 4B, we found a group of cancer types that showed sublinear patterns in the United States and superlinear patterns in Latin America: kidney, colon/rectum, larynx, lymphomas and multiple myelomas, ovary, and pancreas; a second group of cancer types showed a sublinear pattern in the United States but a linear pattern in Latin America (lung and trachea, prostate, melanoma, leukemia, and brain), while esophagus cancer showed a sublinear pattern in both regions. Stomach cancer showed a superlinear pattern in the United States and a linear pattern in Latin America, while liver, uterus, and mouth/oropharynx cancers had a linear pattern in both regions. Last, breast, bladder, cervix, and gallbladder cancer had a superlinear pattern in Latin America and a linear pattern in the United States.

Cardiovascular and other NCDs were clustered into three groups (Fig. 4C). The first group included cirrhosis of the liver, which showed a sublinear pattern in both regions. However, its rank with respect to other NCDs varied by region (Fig. 3), as cirrhosis was the second least sublinear NCD in U.S. cities while being the most sublinear NCD in Latin American cities as a whole, and in Brazilian and Mexican cities. A second group included several other NCDs (oral diseases, musculoskeletal conditions, skin diseases, and sense organ diseases), which had a clear superlinear pattern (and were the most superlinear NCD) in Latin American, Brazilian, and Mexican cities (β = 1.06, 1.09, 1.10, respectively) while having a clear sublinear pattern in U.S. cities (β = 0.90), ranking as the second most sublinear NCD. The last group, including seven of the nine NCDs (cardiovascular, neuropsychiatric, congenital anomalies, diabetes, genitourinary, digestive, and respiratory diseases), were sublinear in U.S. cities and linear in Latin American cities as a whole. However, the ordering of these causes was similar by region, as neuropsychiatric and cardiovascular diseases and congenital anomalies were among the least sublinear NCDs in both regions, while genitourinary, digestive, and respiratory diseases were among the most sublinear NCDs in both regions (Fig. 3). Again, as with other causes of death, these patterns held for Latin American cities as a whole and Brazilian cities, but differed for cities of Mexico, where digestive, genitourinary, and respiratory diseases were among the most superlinear NCDs.

### Injuries

In the case of injury deaths, patterns were mostly consistent between U.S. and Latin American cities (Figs. 3 and 4D). Suicides, road traffic deaths, and other unintentional injuries showed a sublinear pattern in both regions, with a similar magnitude (β = 0.94 and 0.88 for suicides, β = 0.88 and 0.89 for road traffic deaths, and β = 0.94 and 0.96 for other unintentional injuries in U.S. and Latin American cities, respectively). However, their ranking with respect to other causes varied, as they represented some of the most sublinear causes in Latin American cities (including the group of Brazilian cities, Mexican cities, and all other cities excluding Brazil and Mexico), while only road traffic accidents had a similar behavior in the United States, as suicides and other intentional injuries (which include falls and drug overdoses) had an average rank (but were still sublinear). On the other hand, homicides followed a superlinear scaling pattern in both regions (β = 1.12 and 1.10 for U.S. and Latin American cities, respectively). As described above, the only exception to these patterns was for homicides in Mexican and other Latin American cities, which followed a linear pattern [β = 0.97 (95% CI, 0.80 to 1.13) and β = 1.01 (95% CI, 0.91 to 1.12) for Mexican and other Latin American cities, respectively]. Homicides ranked among the most sublinear causes of death for Mexican cities (Fig. 3).

### Secondary analyses

**Fig. 5.**
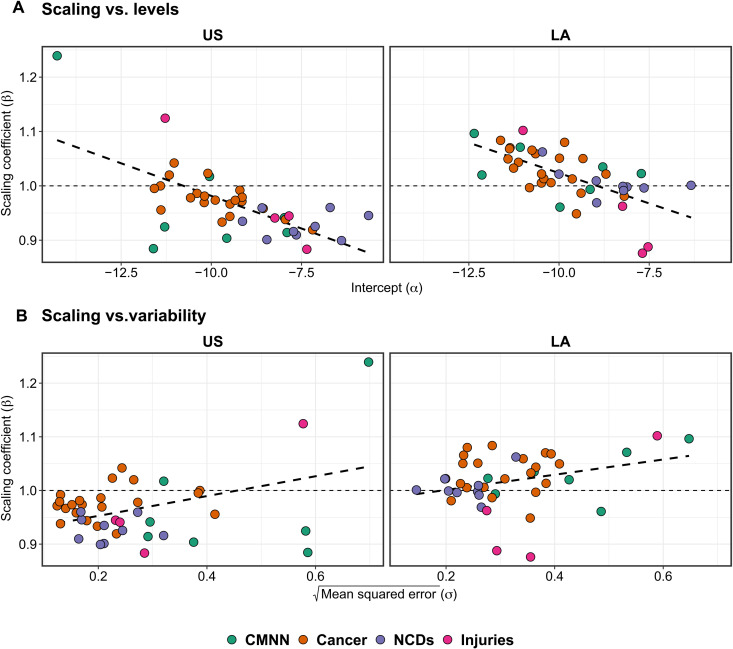
Correlation between scaling exponents and intercepts (levels) and standard deviations (variability) for each cause of death, by region. Coefficients come from the model in Eq. 3, stratified by region.

We also explored the association between the levels and variance of each outcome and their respective scaling coefficient (Fig. 5). We found a negative correlation between scaling exponents and levels (intercepts in the models) (ρ = −0.58 and −0.67 for U.S. and Latin American cities, respectively), indicating that causes of death that are more frequent (higher intercepts) tend to be more sublinear, while causes of death that are less frequent (lower intercepts) tend to be superlinear, similarly across both regions (Fig. 5A). We found a weak positive correlation between scaling exponents and the square root of the mean squared error (ρ = 0.40 and 0.31 for U.S. and Latin American cities, respectively), indicating that causes of death that vary more tend to be more superlinear, while those that have lower variability tend to be more sublinear (Fig. 5B). Sensitivity analyses testing two alternative definitions for U.S. cities, commuting zones and an ad hoc definition based on which counties overlap with the urban area of the city, and adding an indicator to the main model indicating whether the city was the largest in its country all rendered similar results as our main analyses (figs. S2 and S3).

## DISCUSSION

Our analysis of 366 Latin American and 376 U.S. cities (encompassing over 560 million residents) revealed a heterogeneous scaling landscape of mortality across the continent. While in the United States all-cause mortality was relatively lower in larger cities and did not differ by size in Latin American cities, the relation between city size and mortality differed by cause of death and region. Nonviolent injury deaths were sublinear in both regions, whereas homicides and deaths due to STDs/AIDS were superlinear in both regions. NCDs and cancer were generally sublinear (or linear for some cancers) in the United States and mostly linear or superlinear in Latin America. Deaths from CMNN (other than AIDS/STDs) were sublinear in the United States and linear in Latin America.

To date, a number of studies have examined the scaling properties of population health outcomes, most of which focus on single conditions, including STDs and injuries, or cities from a single country. Studies in the United States and Brazil have shown that both HIV/AIDS and STDs scale superlinearly (*4*, *10*, *11*, *13*), consistent with our findings in both the United States and Latin America. In the case of injuries, several studies in the United States and Latin America are consistent with our findings of an overall superlinear scaling pattern of homicides, sublinear scaling pattern of suicides, and sublinear or linear scaling pattern for road traffic injuries (*4*, *14*, *16*, *20*–*24*). Rocha *et al.* (*12*) examined a wider variety of outcomes in U.S. counties, as well as Swedish and Brazilian municipalities, but did not do so in metropolitan areas. Choi *et al.* (*17*) also examined the scaling of mortality by four large NCD groupings in U.S. counties, finding a superlinear pattern for endocrine and metabolic diseases and a sublinear pattern for other NCDs when considering the largest counties.

### Some causes of death show a consistent scaling pattern across regions

The consistency of findings regarding sublinear scaling of nonviolent injury deaths and superlinear scaling of homicides and deaths due to STDs/AIDS across both regions and a diverse set of cities is notable in terms of its consistency, suggesting that some underlying common dynamics may play a role, irrespective of cross-country differences. For example, the sublinear scaling of nonviolent deaths may be because larger cities may have attributes such as more or better public transportation, lower traffic speeds, or sidewalk infrastructure (*25*) that may be linked to lower rates of traffic-related deaths, an important contributor to nonviolent deaths. The sublinear pattern of pedestrian deaths, a component of road traffic deaths, has been reported before in the United States (*16*) and Brazil (*20*), linked to a shorter road traffic network in larger cities (*26*) and higher traffic congestion leading to lower speeds (*16*).

The superlinear scaling of homicides may be linked to differences in social conditions and inequality by city size. Larger cities are often characterized by large inequalities and social exclusion (*27*), originating from both segregation and self-sorting (*28*–*31*) and differential migration to/from cities (*30*, *32*, *33*). On the other hand, larger cities also display wider diversity of economic activities, which may result in improved social and economic outcomes (*32*, *34*). Combinations of some of these factors, along with increased social interactions (*35*), may be linked to higher homicide rates in larger cities, consistent with previous research in the United States and Latin America (*14*). Consistent with these findings, other studies have documented higher rates of crime in larger cities (*4*, *14*), including homicides. The superlinearity of HIV/AIDS and STDs, reported in several previous studies (*4*, *10*, *11*), is likely to emerge from a combination of increased social contacts in larger cities and social, economic, and behavioral differences between cities of different sizes, which leads to increased incidence of both syphilis, gonorrhea, and chlamydia (*10*, *11*) and HIV/AIDS (*4*, *13*).

### Other infectious diseases, and maternal, neonatal, and nutritional conditions display different patterns by region

Deaths from other CMNN conditions were sublinear in the United States and linear or even superlinear in Latin America. This highlights that the distribution of factors driving these conditions is different in both regions. For example, tuberculosis is strongly sublinear in the United States and superlinear in Latin America, especially in Brazilian and other Latin American cities excluding Mexico. Large cities in low and middle income countries show very wide heterogeneity (*36*) in the conditions that give rise to tuberculosis, including poverty and overcrowding, and may also differ in access to high-quality health care services (*37*). In contrast, cities in high-income countries may have better infrastructure. This was shown in a recent study that highlighted that deaths due to conditions for which the health care system can prevent new cases, which include tuberculosis, were especially high in large cities of Latin America (*38*). Migration may be another factor behind the sublinear pattern of tuberculosis mortality in the United States. In 2011, 62% of reported tuberculosis cases in the United States occurred among foreign-born people, mainly Hispanic and Asian individuals (*39*). Barriers to accessing care associated with immigration status can be one of the main factors preventing adherence to treatment, especially among non–English-speaking migrants (*40*). This phenomenon may be more frequent in small and medium-sized cities, given the geographic dispersion of Hispanic immigration (*41*).

### The scaling of NCDs varies widely by specific cause

The different scaling of cancer and NCDs in Latin America (mostly linear) and United States (mostly sublinear) may be related to differential associations of chronic disease risks factors with city size in both regions, particularly considering that most of Latin America has swiftly transitioned toward a predominance of urban areas and the accompanying epidemiological and nutritional transitions that are still in progress (*42*–*44*). Smoking, excessive alcohol consumption, and obesity are more prevalent in smaller compared to larger cities of the United States (*45*). To our knowledge, very few studies have compared NCD risk factor levels across the spectrum of urbanization in Latin America. In an analysis of NCD risk factors by province-level urbanicity in Argentina, Rodríguez López *et al.* (*46*) found that more urbanized provinces had a higher prevalence of smoking but a lower prevalence of hypertension, but these patterns held only for women. Rural to urban comparisons have been more frequent, finding a consistently higher prevalence of NCD risk factors in urban compared to rural areas of Latin America (*47*–*49*). Various environmental and policy features linked to the prevalence of these risks factors [including factors such as air pollution, access to healthier and processed foods, walkability, and green spaces (*50*, *51*)] may be strongly linked to city size in the United States and not so strongly linked to city size in Latin America, as has been observed in a comparison of U.S. and Indian and Chinese cities, which show very different scaling patterns for air pollution (*52*). However, an analysis of our same sample of 366 Latin American cities has shown that larger cities of Latin America have higher levels of air pollution, as measured by PM2.5 levels (*53*). Larger cities of both regions may also have higher health care capacity (*54*–*58*), although a recent study in Latin American cities showed that deaths due to conditions for which the health care system should prevent cases are more frequent in larger cities, while deaths due to chronic conditions requiring substantial care are lower in larger cities (*38*).

The mostly sublinear scaling observed for cancers in the United States could also reflect better cancer survival in larger cities (*59*) as cancer mortality rates are especially sensitive to survival differences. Diagnostic capabilities may also be associated with city size, and these associations may be different in the United States and Latin America. We explored the scaling properties of ill-defined deaths, a marker of quality of coding of causes of death and potentially to improved diagnostic capabilities, and found them to be similarly sublinear in both regions (β = 0.95).

### Implications

Our findings have implications for understanding the phenomenon of urban scaling and for prevailing urban scaling theories, which see the phenomenon as driven by common universal mechanisms rather than place-specific effects. The inclusion of cities in 10 different countries of Latin America and the United States and the study of multiple causes of death allowed us to explore heterogeneities by region and cause of death. Although we found some commonalities, we also observed important differences in the scaling behavior of specific causes of death and, in some cases, in the scaling behavior of a given cause of death across regions or countries. These differences were reflected in different values of scaling coefficients, their rankings within country or region, or both. We posit three possible explanations for these patterns.

First, and as outlined in the previous sections, the associations of health-relevant city characteristics including social, environmental, health care–related, and behavioral factors with city size may differ by domain and by region, and these features may relate differently to different causes of death. In other words, because different processes link city size to different health outcomes and the links between the drivers of these processes and city size may additionally vary by region, it is not surprising that the scaling of mortality varies by specific cause and region.

Second, and as proposed by Arcaute *et al.* (*60*) and Pumain (*61*), scaling patterns may be affected by path dependencies (historical contingencies) that influence specific features of cities regardless of their size (e.g., the role of San Francisco as a technological hub, despite its relatively small size compared to other cities). As a result, the context in which each city grew to its current size may affect the consequences of that growth and, therefore, the currently observed relationship between city size and mortality. Relatedly, Jedwab and Vollrath (*62*) have proposed a model of megacity growth, where they posit that current features of megacities vary depending on whether these cities grew before or after the transition to the current low mortality regime.

Third, as suggested by Gomez-Lievano *et al.* (*32*), scaling may also be a function of the nature of multifactorial causal processes at work. According to Gomez-Lievano *et al.* (*32*), outcomes that require the presence of multiple different factors (and that are therefore less common and have more variability) are more likely to occur in larger cities (behave superlinearly) because these multiple factors are more likely to co-occur in larger cities. Consistent with this hypothesis, we found that less common and more variable causes of death tended to be more superlinear. However, these associations do not necessarily prove the theory as, for example, it is not immediately obvious that homicide and STD/AIDS deaths (strongly superlinear in our data) require more factors to co-occur than nonviolent injury deaths (which were strongly sublinear).

The stage of the epidemiological transition (*63*) of each country could affect scaling of health through each of the three explanations for scaling behaviors that we describe: through context and outcome-specific processes, through their histories and path dependencies, and by altering the relative prevalence of outcomes that require multiple factors to occur. The heterogeneity in stages of the epidemiologic transition reflected in countries we analyzed (*64*–*66*) could thus also explain heterogeneities in scaling coefficients across regions and countries.

Regardless of the processes that drive the scaling phenomena we observed, these relations have implications for public health interventions and urban policy. Knowing whether certain health outcomes scale with city size, and how, may allow for more precise resource allocation. For example, if two diseases show opposing scaling patterns, resources to prevent one with superlinear scaling could be focused on larger cities, while resources to prevent one with sublinear scaling could be focused on smaller cities. Moreover, a greater understanding of the drivers of the scaling phenomena could provide insights on whether there is an optimal city size (an important consideration for urban policy), a topic that has been extensively studied from a productivity perspective (*67*, *68*), but rarely analyzed for population health optimization (*69*).

### Caveats and limitations

There are some concerns regarding the quality of mortality data obtained from vital registration. First, there is a known underregistration of death counts that varies by country and subnationally (*65*, *70*), which we addressed by applying an ensemble of state-of-the-art demographic methods to address this at the city level for Latin American cities, as reported elsewhere (*65*, *71*). Second, a number of deaths are coded using ill-defined causes [International Classification of Diseases International Classification of Diseases (10th version) (ICD-10) codes R00 to R94 and R96 to R99], which we addressed by redistributing ill-defined deaths into more specific categories, based on age, sex, country, and year, as done in other studies (*65*). Ill-defined diseases scaled sublinearly in both U.S. cities and Latin American cities (β = 0.95), indicating improved coding of causes of death in larger cities, as compared to smaller cities, although the CIs crossed linearity in both cases (95% CI, 0.89 to 1.02 and 0.87 to 1.03 in U.S. and Latin American cities, respectively). Moreover, the coding of causes of death, and our grouping of causes of death, also has limitations. For example, our categorization of deaths due to communicable, maternal, neonatal, and nutritional conditions proved to be highly heterogeneous, as an analysis with more detailed causes of death showed very different scaling coefficients. This classification is rooted in the idea of epidemiologic transitions (*63*), which has been criticized (*72*) for, among other things, ignoring the potential emergence of new infectious diseases [such as HIV/AIDS or, more recently, coronavirus disease 2019 (COVID-19)]. Improvements in the categorization of causes of death may provide for more consistent results of the scaling patterns of deaths by cause.

A third caveat to our findings is related to the sensitivity of urban scaling properties to the definition of what constitutes a city and its boundaries (*60*). However, our sensitivity analysis exploring three alternative definitions for U.S. cities and our addition of an adjustment covariate for the largest city in each country [to control for the “dragon-king” cities described by Arcaute *et al.* (*60*)] showed no changes to scaling patterns. Fourth, we are analyzing data for 11 countries, with their own regional differences in their urban systems. Historical factors have influenced the population and characteristics of cities in each country, resulting in potential challenges comparing cities of the same size across different countries. For example, job opportunities and the most advanced health care facilities in each country tend to concentrate in their largest cities (*54*–*58*). However, the size of these largest cities varies widely by country: The largest cities in the United States, Brazil, Mexico, and Argentina are all above 15 million, the largest cities in Chile, Colombia, and Peru have between 6 and 10 million residents, while the largest cities in Costa Rica, Guatemala, El Salvador, and Panama are all below 3.2 million residents. Therefore, if the likelihood of death increases (or decreases) with city size, as size approaches the largest city in a country, cities in smaller countries may not be comparable to similarly sized cities in larger countries. We controlled our main analysis for country, by adding a dummy covariate specific to each country, but we cannot rule out regional differences in each country that may not be captured by this adjustment. Last, our results are drawn from a cross-sectional sample and should not be interpreted directly as a measure of how much a city should grow (or shrink) to achieve that optimal size. That assertion assumes that cross-sectional associations reflect longitudinal associations and also requires the assumption of ergodicity, or lack of path dependence (*61*), which assumes that a given outcome depends only on the current state of the city, with no consideration to the path it took to get there, which may (*73*) or may not hold for cities (*61*, *74*). Future studies should leverage longitudinal data that allow for the exploration of how city features change over time.

As the world continues urbanizing and as the COVID-19 pandemic brings scrutiny to the assumption that urban living translates into population-wide benefits, including better health profiles, it is especially important to deepen our understanding of how city size is related to health. Sublinear scaling of some causes of death indicates that larger cities can benefit from efficiency in services, from educational and job opportunities, and from environments and policies that may promote health. In contrast, superlinear scaling highlights the potentially negative correlates of larger cities, such as crowding, pollution, violence, and inequality.

Our results characterizing a comprehensive set of mortality patterns across a wide range of cities in the entire region of the Americas support the idea that there is no unique relation between city size and different health conditions, and that the application of urban scaling theories, which see the phenomenon as driven by common universal mechanisms (*4*, *5*), to health outcomes, may need to be adapted to explain this heterogeneity. The inclusion of a large number of Latin American cities in our study advances the field by exploring the phenomenon of urban scaling in countries at varying income levels. Our findings raise questions about how city attributes can be leveraged to be health promoting while minimizing any adverse consequences. Understanding the processes that explain the heterogeneity in scaling behavior of mortality that we observed could be useful for health and urban policy.

It is possible that the ability of cities to maximize beneficial health, social, physical, and service environments declines above a certain size. Identifying the presence of a tipping point and understanding the historically and socially situated factors that drive its location are crucial for planning purposes and for public health preparedness and responsiveness. This task requires considering health, energy efficiency, environmental, and economic impacts, much in line with the global interconnected targets set forth as the Sustainable Development Goals. An optimal city size could be an important universal social goal, minimizing adverse environmental impacts, maximizing health benefits, and sustaining an increased level of creativity and innovation that has long been a major characteristic of cities and the reason for its success to become the preferred shelter for the human population.

## MATERIALS AND METHODS

### Study setting

We used data on 366 Latin American and 376 U.S. cities. Latin American cities were defined as urban agglomerations of administrative units (municipios, comunas, distritos, partidos, etc.) that overlapped with the urban extent of the city, in 10 countries (*65*, *71*): Argentina, Brazil, Chile, Colombia, Costa Rica, El Salvador, Mexico, Peru, and Panama. U.S. cities were defined as core-based statistical areas or the agglomeration of counties adjacent (and connected through commuting patterns) or part of a core area with at least 10,000 people. To make analyses comparable across both settings, we restricted our analysis to Latin American and U.S. cities with more than 100,000 people in 2010. We pooled data for the 2012–2016 period except for El Salvador (2010–2014), due to data availability.

### Data sources

Data for Latin America were obtained from the Salud Urbana en America Latina (SALURBAL) study, which has compiled and harmonized vital registration and other health data (*65*, *71*). Data for the United States were obtained from the National Vital Statistics System (*75*) and the Census Bureau. In all cases, we obtained all mortality records for the time frame of the study georeferenced to the county or county-equivalent level, with data on cause of death. We also obtained intercensal population estimations or postcensal projections by county or county-equivalent and age.

### Variables

The main exposure investigated was the average yearly city population size in the period of the study, henceforth referred as city size. The main outcomes investigated are average yearly mortality counts by cause by city of residence. We classified causes of death based on the categories of the Global Health Estimates classification (*76*). Causes of death were first divided into six large groupings, three groupings of diseases, and three groupings of external causes (injuries): (i) CMNN, (ii) cancer, (iii) cardiovascular disease and other NCDs (CVD/NCDs), (iv) nonviolent injuries (road traffic accidents and other unintentional injuries), (v) suicides, and (vi) homicides. We also further divided these categories into 41 fine-grained groups. Table S1 contains details on the groupings and corresponding codes of ICD-10.

We addressed three critical challenges of vital registration data. First, we imputed missing age and sex (0.2% and 0.05% of deaths in Latin American cities and 0.005% and 0% of deaths in U.S. cities had missing information on age and sex, respectively) using a pro rata redistribution, based on cause of death, sex or age, country, and year. Second, we redistributed deaths assigned to ill-defined causes (4.1% for Latin American cities and 1.5% for U.S. cities) using a pro rata redistribution by age, sex, country, and year. Ill-defined causes are causes of death that do not provide useful information on the cause of death for public health purposes, and we define these as ICD-10 codes R00-R94 and R96-R99. Third and last, we corrected for the underregistration of deaths at the city level by using an ensemble of death distribution methods. We assumed that U.S. cities had complete coverage of death counts and conducted this correction only for Latin American cities. More details on these three steps are available elsewhere (*65*). We also adjusted for the compositional effect of different age structures across cities, which have been shown to drive some scaling patterns (*18*) and which have a strong effect on mortality patterns. For this, we calculated the proportion of city residents aged 0 to 14, 15 to 39, 40 to 64, and 65 years and above, for the years of the study.

### Statistical analysis

The basic scaling model (*4*) used for all analyses is a nonlinear power law relationship of the form

$$Y=Y_{0}*N^{\beta }$$ (1)

We estimate these parameters by taking the natural logarithm of both sides of Eq. 1 and estimating an ordinary least squares (OLS) regression of the form

$$\text{ln}\;(Y_{\mathrm{ij}})=\alpha +\beta *\text{ln}\;(N_{\mathrm{ij}})+\epsilon_{\mathrm{ij}}$$ (2)

where *Y_ij_* is the number of deaths for the *i*th city in the *j*th country, α is a constant, β is the scaling exponent (β < 1 representing sublinear scaling, β > 1 superlinear scaling, and β = 1 linear scaling), *N_ij_* is the population of the city, and ε is a residual (*4*). Data from different countries may present the same scaling pattern (same exponent β) but different magnitude (different α). Thus, to account for possible different levels of mortality rates and coding of causes of death by country, and to control for the role of age distribution in mortality, we also expanded the model in Eq. 2, based on previous work (*33*), as

$$\begin{array}{c} \text{log}\;(Y_{\mathrm{ij}})=\alpha +\beta *\text{log}(N_{\mathrm{ij}})+\alpha_{2}*{\text{Country}}_{j}+\alpha_{3}*\text{Prop}{\left(15\_39\right)}_{\mathrm{ij}}+\\ \alpha_{4}*\text{Prop}{\left(40\_64\right)}_{\mathrm{ij}}+\alpha_{5}*\text{Prop}{\left(65p\right)}_{\mathrm{ij}}+\epsilon_{\mathrm{ij}} \end{array}$$ (3)

Here, the vector of variables Country_j_ refers to the country where the city is located, and Prop(15_39), Prop(40_64), and Prop(65p) represent the percentage of the population in each city aged 15 to 39, 40 to 64, and 65+. We ran the model in Eq. 3 for all cities combined and also stratified these models by region to separately obtain scaling exponents (β) and intercepts (α) values for Latin American and U.S. cities separately. Last, we also leveraged the large number of cities in two Latin American countries to estimate country-specific scaling coefficients in Brazil (*n* = 152 cities), Mexico (*n* = 92), and all other Latin American cities (*n* = 122). Models for a single country (i.e., United States, Brazil, and Mexico) do not include any adjustment for country, while models for multiple countries (i.e., the models for all Latin American countries and for all Latin American countries minus Brazil and Mexico) include the adjustment covariates for country.

Last, to explore whether the general level and variability of each type of cause of death was associated with its scaling behavior, we followed the approach of Gomez-Lievano *et al.* (*32*). For this, we compared the scaling exponents (β) for each cause of death with the following: (i) their corresponding intercepts (α), which are a metric of the general levels of the phenomenon, and (ii) the square root of the mean squared error, which are a metric of the variability of the phenomenon after accounting for city size, both obtained from Eq. 3 above. We explored the correlation between scaling patterns and general levels and variability by region, both graphically and by calculating Pearson’s correlation coefficient.

We carried two sensitivity analyses. First, to test whether city definitions altered the scaling properties of causes of death, we explored two alternative definitions in the United States: (i) commuting zones (*77*) and (ii) an ad hoc definition designed to mimic the SALURBAL city definition (*65*, *71*). This ad hoc definition was obtained by overlaying U.S. urban areas (block-level definition of urbanized areas) with U.S. counties and defining cities as agglomerations of counties that overlay with the city urbanized area. Details on these city definitions are available in table S6. Second, given the heterogeneity of city sizes across countries (*78*) and the potential differential importance of capital cities as economic hubs (*60*), we tested whether adding a covariate to Eq. 3 indicating whether the city was the largest in its country changed the inferences we observed in our main analysis. All analyses were conducted in R v4.1.

## Notes

View/request a protocol for this paper from *Bio-protocol*.
