# Supplementary material for: Scaling of mortality in 742 metropolitan areas of the Americas
Source: Sci Adv. Author manuscript; Available in PMC 2021 Dec 27. (PMC8654292; doi:10.1126/sciadv.abl6325)
Supplement: Supplementary materials [file EMS140651-supplement-Supplementary_materials.pdf]

Science Advances
AAAS

Supplementary Materials for

Scaling of mortality in 742 metropolitan areas of the Americas

Usama Bilal*, Caio P. de Castro, Tania Alfaro, Tonatiuh Barrientos-Gutierrez, Mauricio L. Barreto, Carlos M. Leveau, Kevin Martinez-Folgar, J. Jaime Miranda, Felipe Montes, Pricila Mullachery, Maria Fatima Pina, Daniel A. Rodriguez, Gervasio F. dos Santos, Roberto F. S. Andrade, Ana V. Diez Roux

*Corresponding author. Email: ubilal@drexel.edu

Published 8 December 2021, *Sci. Adv.* **7**, eabl6325 (2021)
DOI: 10.1126/sciadv.abl6325

This PDF file includes:

Figs. S1 to S3
Tables S1 to S6

Supplementary Table S1: International Classification of Diseases version 10 (ICD-10) codes.

Category	ICD10 codes
CMNN	A00-B99, G00-G04, G14, N70-N73, J00-J06, J09-J18, J20-J22, H65-H66, O00-O99, P00-P96, E00-E02, E40-E46, E50, D50-D53, D64.9, E51-E64
Cancer	C00-C97, D00-D48
CVD/NCDs	D55-D64 (- D64.9) D65-D89, E03-E07, E10-E16, E20-E34, E65-E88, F01-F99, G06-G98 (- G14), H00-H61, H68-H93, I00-I99, J30-J98, K00-K92, N00-N64, N75-N98, L00-L98, M00-M99, Q00-Q99, X41, X42, X45, R95
Unintentional injuries	V01-X59 (- X41, X42, X45), Y35, Y36, Y40-Y86, Y88, Y89
Suicides	X60-X84, Y870
Homicides	X85-Y09, Y871
Ill-defined diseases	R00-R94, R96-R99
Injuries of ill-defined intent	Y10-Y34, Y872
Tuberculosis	A15-A19, B90
STDs and HIV/AIDS	A50-A64, N70-N73
Respiratory Infections	J00-J06, J09-J18, J20-J22, H65-H66
Other Infectious Diseases	A00-B99 (- A15-A19, A50-A64, B90), G00, G03-G04, G14
Maternal Conditions	O44-O46, O67, O72
Perinatal Conditions	P00-P96
Nutritional Conditions	E00-E02, E40-E46, E50, D50-D53,D64.9, E51-E64
Mouth and oropharynx cancers	C00-C14
Oesophagus cancer	C15
Stomach cancer	C16
Colon and rectum cancers	C18-C21
Liver cancer	C22
Pancreas cancer	C25
Trachea, bronchus, lung cancers	C33-C34
Melanoma and other skin cancers	C43-C44
Breast cancer	C50
Cervix uteri cancer	C53
Corpus uteri cancer	C54-C55
Ovary cancer	C56
Prostate cancer	C61
Kidney and ureter cancer	C64-C66
Bladder cancer	C67
Brain and nervous system cancers	C70-C72
Gallbladder and biliary tract cancer	C23-C24
Larynx cancer	C32
Lymphomas, multiple myeloma	C81-C90, C96
Leukaemia	C91-C95
Other Cancers	C62, C45, C73, C17, C26-C31, C37-C41, C46-C49, C51,C52, C57-C60, C63, C68, C69, C74- C80, C97, D00-D48
Diabetes mellitus and other endocrine disorders	E10-E14, D55-D63, D65-D89, E03-E07, E15-E16, E20-E34, E65-E88
Neuropsychiatric disorders	F01-F99, G06-G98 (-G14), X41, X42, X45
Cardiovascular diseases	I00-I99
Respiratory diseases	J30-J98
Digestive Diseases	K20-K92 (except K70, K74)
Cirrhosis of the liver	K70, K74
Genitourinary diseases	N00-N64, N75-N98
Congenital anomalies	Q00-Q99
Other NCDs	H00-H61, H68-H93, L00-L98, M00-M99, K00-K14, R95
Road Traffic Accidents	V01-V04, V06, V09-V80, V87, V89, V99
Other Unintentional Injuries	V05, V07, V08, V81-V86, V88, X01-X59 (-X41, X42, X45), Y40-Y86, Y88, Y89
Suicides	X60-X84
Violence	X85-Y09, Y35-Y36, Y87

Footnote: classification based on the Global Health Estimates study.

Supplementary Figure S1: Scaling of All-Cause and Cause-Specific Mortality relative to population in US and Latin American Cities, adjusted by age distribution and country.

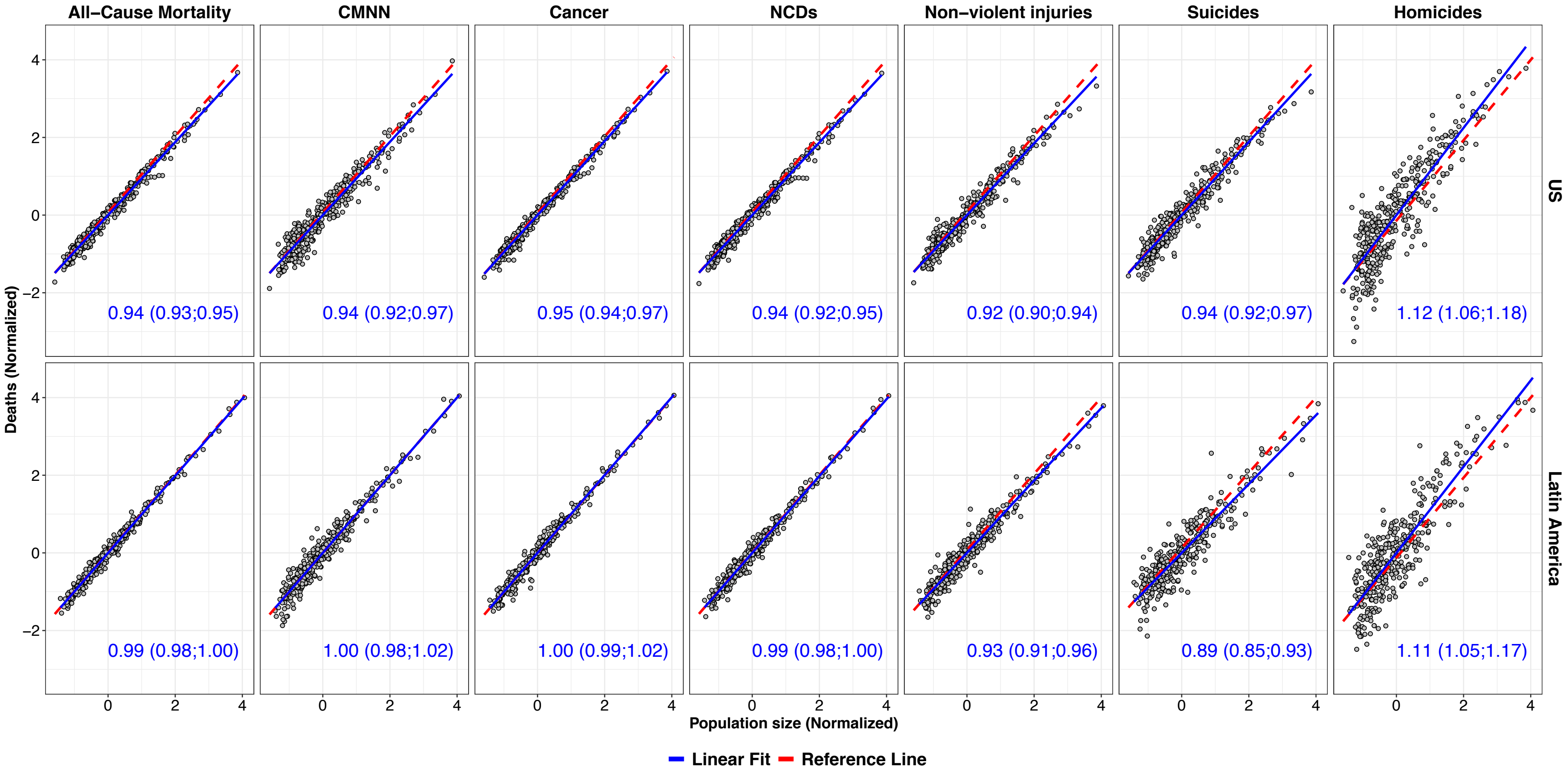

Footnote: solid blue lines are linear fits of log(deaths) on log(population), adjusted by country and age distribution, red dashed line are reference lines (B=1). Coefficients (95% CI) are adjusted coefficients of log(deaths) on log(population), adjusted by country and age distribution, stratified by region. CMNN=communicable, maternal, neonatal and nutritional conditions. NCDs=non-communicable diseases.

Supplementary Table S2: coefficients of determination (R^2) by cause of death for all US and Latin American cities.

Cause/group	Unadjusted	Adjusted*	US**	LA**	BR**	MX**	LA (no MX/BR)**
**All-Cause Mortality**	91.9%	98.5%	98.4%	99.0%	99.3%	98.9%	99.0%
**CMNN**	85.8%	95.1%	94.2%	95.9%	96.1%	97.0%	96.4%
**Cancer**	83.1%	98.6%	98.8%	98.5%	98.7%	98.5%	98.3%
**CVD/NCDs**	87.6%	98.1%	98.1%	98.7%	99.0%	98.9%	98.6%
**Non-violent injuries**	93.3%	95.3%	96.0%	94.9%	96.0%	95.4%	93.3%
**Suicides**	67.5%	92.2%	94.2%	88.1%	89.0%	86.3%	88.9%
**Homicides**	56.0%	85.9%	81.0%	82.8%	86.5%	64.6%	88.8%

Footnote: CMNN: communicable, maternal, neonatal and nutritional diseases. CVD/NCDs: cardiovascular disease and other non-communicable diseases. * Adjusted model is adjusted by age structure and country. **Stratified models are run only on the indicated sample (e.g. LA is ran with all LA cities), all adjusted by age structured and country (where relevant). For the Latin American Cities part, All Cities includes the 366 cities in 10 countries, while BR includes Brazilian cities (n=152), MX includes Mexican cities only (n=92), and All minus BR/MX includes all LA cities except for those in BR and MX (n=122). BR: Brazil. MX: Mexico.

Supplementary Table S3: scaling coefficients (β, 95% CI) by detailed cause of death

Cause	US**	LA**	BR**	MX**	LA (- BR/MX)**
Tuberculosis	0.88 (0.82;0.95)	1.10 (1.02;1.17)	1.18 (1.10;1.27)	0.83 (0.63;1.03)	1.09 (0.97;1.21)
STDs and HIV/AIDS	1.24 (1.17;1.31)	1.07 (1.01;1.13)	1.07 (1.00;1.13)	0.90 (0.75;1.04)	1.17 (1.06;1.28)
Infectious Diseases	0.94 (0.91;0.97)	1.03 (0.99;1.08)	1.03 (0.98;1.09)	1.04 (0.98;1.11)	1.03 (0.94;1.11)
Respiratory Infections	0.91 (0.88;0.94)	1.02 (0.99;1.05)	1.00 (0.95;1.05)	1.10 (1.04;1.17)	1.02 (0.98;1.07)
Maternal Conditions	0.92 (0.86;0.99)	1.02 (0.97;1.07)	1.09 (1.02;1.17)	0.99 (0.91;1.06)	0.95 (0.86;1.04)
Perinatal Conditions	1.02 (0.98;1.05)	0.99 (0.96;1.03)	1.01 (0.97;1.05)	0.97 (0.90;1.03)	0.99 (0.92;1.06)
Nutritional Conditions	0.90 (0.87;0.94)	0.96 (0.91;1.02)	0.95 (0.86;1.03)	0.94 (0.83;1.05)	1.01 (0.92;1.11)
Mouth and oropharynx cancers	0.98 (0.95;1.01)	1.03 (0.99;1.07)	1.02 (0.97;1.06)	1.02 (0.92;1.13)	1.06 (0.98;1.14)
Oesophagus cancer	0.93 (0.91;0.95)	0.95 (0.91;0.99)	0.94 (0.89;1.00)	0.98 (0.91;1.05)	0.93 (0.85;1.00)
Stomach cancer	1.04 (1.02;1.07)	0.99 (0.95;1.02)	1.03 (0.99;1.07)	0.98 (0.92;1.04)	0.93 (0.86;0.99)
Colon and rectum cancers	0.96 (0.94;0.97)	1.05 (1.02;1.08)	1.07 (1.04;1.11)	1.05 (0.99;1.10)	1.02 (0.98;1.07)
Liver cancer	1.02 (1.00;1.05)	1.01 (0.98;1.04)	1.02 (0.98;1.06)	0.97 (0.88;1.05)	1.00 (0.95;1.05)
Pancreas cancer	0.98 (0.97;0.99)	1.05 (1.02;1.08)	1.04 (1.00;1.08)	1.09 (1.03;1.15)	1.05 (0.99;1.11)
Trachea, bronchus, lung cancers	0.92 (0.90;0.94)	1.02 (0.99;1.06)	1.01 (0.97;1.05)	1.00 (0.91;1.09)	1.04 (0.97;1.10)
Melanoma and other skin cancers	0.97 (0.95;0.99)	1.00 (0.96;1.04)	1.01 (0.95;1.07)	1.02 (0.95;1.10)	0.98 (0.90;1.06)
Breast cancer	0.99 (0.98;1.01)	1.08 (1.05;1.11)	1.09 (1.05;1.12)	1.05 (0.99;1.10)	1.08 (1.03;1.13)
Cervix uteri cancer	1.00 (0.96;1.04)	1.07 (1.02;1.11)	1.14 (1.06;1.21)	0.95 (0.89;1.01)	1.00 (0.94;1.07)
Corpus uteri cancer	1.02 (0.99;1.05)	1.01 (0.97;1.06)	1.03 (0.96;1.10)	1.05 (0.96;1.13)	0.97 (0.90;1.04)
Ovary cancer	0.98 (0.96;1.00)	1.08 (1.05;1.12)	1.11 (1.06;1.16)	1.10 (1.03;1.16)	1.04 (0.98;1.09)
Prostate cancer	0.97 (0.96;0.99)	1.01 (0.99;1.04)	1.00 (0.97;1.04)	1.02 (0.96;1.08)	1.01 (0.96;1.05)
Other Cancer	0.94 (0.92;0.95)	0.98 (0.96;1.00)	0.96 (0.92;1.00)	1.02 (0.98;1.05)	0.99 (0.94;1.03)
Kidney and ureter cancer	0.94 (0.93;0.96)	1.06 (1.02;1.10)	1.03 (0.98;1.09)	1.06 (0.99;1.13)	1.09 (1.02;1.17)
Bladder cancer	0.99 (0.97;1.01)	1.07 (1.03;1.11)	1.05 (1.00;1.10)	1.10 (1.00;1.20)	1.08 (0.99;1.16)
Brain and nervous system cancers	0.97 (0.96;0.99)	1.01 (0.98;1.03)	0.99 (0.96;1.02)	1.01 (0.95;1.07)	1.01 (0.95;1.07)
Gallbladder and biliary tract cancer	1.00 (0.96;1.03)	1.04 (1.00;1.08)	1.10 (1.04;1.15)	1.05 (0.97;1.12)	0.98 (0.90;1.06)
Larynx cancer	0.96 (0.91;1.00)	1.05 (1.00;1.10)	1.05 (0.99;1.10)	0.97 (0.87;1.07)	1.10 (1.00;1.20)
Lymphomas, multiple myeloma	0.97 (0.96;0.98)	1.07 (1.04;1.09)	1.08 (1.04;1.12)	1.06 (1.00;1.11)	1.06 (1.02;1.11)
Leukaemia	0.97 (0.95;0.98)	1.02 (1.00;1.04)	1.01 (0.98;1.04)	1.03 (0.98;1.08)	1.03 (0.99;1.07)
Diabetes mellitus	0.93 (0.90;0.95)	1.00 (0.97;1.03)	0.98 (0.93;1.03)	1.02 (0.97;1.07)	1.00 (0.96;1.05)
Neuropsychiatric disorders	0.96 (0.94;0.98)	1.01 (0.98;1.04)	1.01 (0.98;1.05)	1.04 (0.98;1.09)	1.00 (0.93;1.06)
Other NCD	0.90 (0.88;0.92)	1.06 (1.03;1.10)	1.09 (1.04;1.14)	1.10 (1.05;1.15)	1.02 (0.94;1.10)
Cardiovascular diseases	0.95 (0.93;0.96)	1.00 (0.98;1.02)	1.00 (0.98;1.02)	1.01 (0.97;1.04)	0.99 (0.96;1.02)
Respiratory diseases	0.90 (0.88;0.92)	1.00 (0.97;1.02)	0.97 (0.94;1.00)	1.04 (0.99;1.09)	1.00 (0.95;1.05)
Digestive Diseases	0.91 (0.89;0.93)	1.00 (0.98;1.02)	1.01 (0.98;1.04)	1.06 (1.02;1.11)	0.94 (0.90;0.99)
Cirrhosis of the liver	0.96 (0.93;0.99)	0.97 (0.94;1.00)	0.96 (0.93;1.00)	0.98 (0.91;1.06)	1.00 (0.94;1.05)
Genitourinary diseases	0.92 (0.88;0.95)	0.99 (0.96;1.02)	0.99 (0.95;1.03)	1.05 (1.00;1.10)	0.97 (0.91;1.02)
Congenital anomalies	0.93 (0.91;0.96)	1.02 (1.00;1.04)	1.02 (0.99;1.04)	1.04 (1.00;1.08)	1.02 (0.97;1.07)
Road traffic accidents	0.88 (0.85;0.91)	0.89 (0.86;0.92)	0.89 (0.85;0.92)	0.92 (0.84;1.01)	0.85 (0.79;0.91)
Other unintentional injuries	0.94 (0.92;0.97)	0.96 (0.93;0.99)	0.99 (0.95;1.03)	0.96 (0.90;1.02)	0.93 (0.87;0.99)
Suicide	0.94 (0.92;0.97)	0.88 (0.84;0.92)	0.88 (0.83;0.93)	0.91 (0.82;1.00)	0.87 (0.79;0.95)
Violence	1.12 (1.07;1.18)	1.10 (1.04;1.17)	1.17 (1.09;1.25)	0.97 (0.80;1.13)	1.01 (0.91;1.12)

Footnote: models are run only on the indicated sample (e.g. LA is ran with all LA cities), all adjusted by age structured and country (where relevant). For the Latin American Cities part, All Cities includes the 366 cities in 10 countries, while BR includes Brazilian cities (n=152), MX includes Mexican cities only (n=92), and All minus BR/MX includes all LA cities except for those in BR and MX (n=122). BR: Brazil. MX: Mexico.

Supplementary Table S4: coefficients of determination (R^2) by detailed cause of death

Cause	US**	LA**	BR**	MX**	LA (-BR/MX)**
Tuberculosis	72.4%	74.5%	85.0%	59.8%	80.9%
STDs and HIV/AIDS	77.1%	81.4%	87.8%	76.2%	83.6%
Infectious Diseases	91.8%	89.5%	90.5%	93.0%	87.7%
Respiratory Infections	91.6%	93.8%	92.0%	93.7%	96.2%
Maternal Conditions	74.5%	85.6%	85.8%	89.8%	85.3%
Perinatal Conditions	92.2%	92.6%	95.3%	91.9%	90.3%
Nutritional Conditions	86.2%	81.7%	77.9%	80.1%	83.1%
Mouth and oropharynx cancers	93.3%	91.5%	94.2%	85.6%	89.2%
Oesophagus cancer	96.1%	91.1%	88.5%	91.4%	90.7%
Stomach cancer	95.2%	92.9%	93.8%	93.7%	91.4%
Colon and rectum cancers	97.5%	95.9%	95.7%	94.8%	96.9%
Liver cancer	95.6%	93.4%	95.7%	87.7%	94.8%
Pancreas cancer	98.4%	94.7%	96.0%	94.5%	94.2%
Trachea, bronchus, lung cancers	94.5%	92.3%	94.5%	88.2%	92.7%
Melanoma and other skin cancers	95.9%	88.8%	88.7%	90.2%	88.5%
Breast cancer	98.4%	95.6%	96.4%	95.1%	95.7%
Cervix uteri cancer	87.8%	88.3%	87.5%	92.8%	90.8%
Corpus uteri cancer	94.2%	88.4%	85.5%	89.2%	91.1%
Ovary cancer	97.4%	93.9%	93.5%	94.8%	94.1%
Prostate cancer	97.8%	95.1%	95.9%	93.6%	95.9%
Other Cancer	98.2%	95.8%	94.5%	97.9%	96.4%
Kidney and ureter cancer	96.8%	92.0%	90.7%	92.6%	92.5%
Bladder cancer	96.2%	90.2%	92.1%	87.0%	90.1%
Brain and nervous system cancers	97.2%	95.0%	96.8%	93.9%	93.5%
Gallbladder and biliary tract cancer	88.0%	90.4%	90.7%	90.6%	89.6%
Larynx cancer	85.4%	88.7%	91.3%	86.4%	86.2%
Lymphomas, multiple myeloma	98.5%	95.6%	95.3%	96.1%	96.1%
Leukaemia	98.1%	96.4%	96.2%	96.4%	96.8%
Diabetes mellitus	93.9%	95.1%	92.3%	95.2%	95.3%
Neuropsychiatric disorders	97.3%	94.9%	96.3%	95.2%	93.5%
Other NCD	95.2%	92.3%	93.1%	96.0%	90.4%
Cardiovascular diseases	97.1%	98.0%	98.6%	98.0%	97.7%
Respiratory diseases	95.5%	95.4%	95.9%	95.5%	95.4%
Digestive Diseases	97.1%	96.1%	97.4%	96.6%	95.2%
Cirrhosis of the liver	92.9%	94.2%	95.8%	90.6%	94.1%
Genitourinary diseases	90.1%	93.6%	93.4%	96.3%	93.6%
Congenital anomalies	95.7%	96.5%	97.6%	97.2%	94.2%
Road traffic accidents	91.3%	90.3%	94.1%	84.9%	89.8%
Other unintentional injuries	94.7%	92.7%	93.8%	93.3%	91.3%
Suicide	94.2%	88.1%	89.0%	86.3%	88.9%
Violence	81.0%	82.8%	86.5%	64.6%	88.8%

Footnote: models are run only on the indicated sample (e.g. LA is ran with all LA cities), all adjusted by age structured and country (where relevant). For the Latin American Cities part, All Cities includes the 366 cities in 10 countries, while BR includes Brazilian cities (n=152), MX includes Mexican cities only (n=92), and All minus BR/MX includes all LA cities except for those in BR and MX (n=122). BR: Brazil. MX: Mexico.

Supplementary Table S5: Scaling patterns of detailed causes of death, by type of pattern, cause, and region

			Latin American Cities			
**Cause**	**Pattern**	**US Cities**	**All Cities****	**BR****	**MX****	**All minus BR/MX****
CMNN	Sublinear	5	0	0	0	0
CMNN	Linear	1	5	4	6	6
CMNN	Superlinear	1	2	3	1	1
Cancer	Sublinear	13	1	1	0	1
Cancer	Linear	7	10	13	18	17
Cancer	Superlinear	1	10	7	3	3
NCDs	Sublinear	9	1	1	0	1
NCDs	Linear	0	7	7	6	8
NCDs	Superlinear	0	1	1	3	0
Injuries*	Sublinear	3	3	2	1	3
Injuries*	Linear	0	0	1	3	1
Injuries*	Superlinear	1	1	1	0	0

Footnote: a pattern is defined as sublinear if $\beta<1$ and the 95% confidence interval does not cover 1; as superlinear if $\beta>1$ and the 95% confidence interval does not cover 1; and as linear otherwise (confidence interval covers 1). Models are adjusted by age distribution and country. *Injuries include non-violent injuries, suicides, and homicides. **For the Latin American Cities part, All Cities includes the 366 cities in 10 countries, while BR includes Brazilian cities (n=152), MX includes Mexican cities only (n=92), and All minus BR/MX includes all LA cities except for those in BR and MX (n=122).

Supplementary Figure S2: comparison of scaling coefficients using core-based statistical areas (CBSAs, main analysis) vs commuting zones or urban areas in US cities

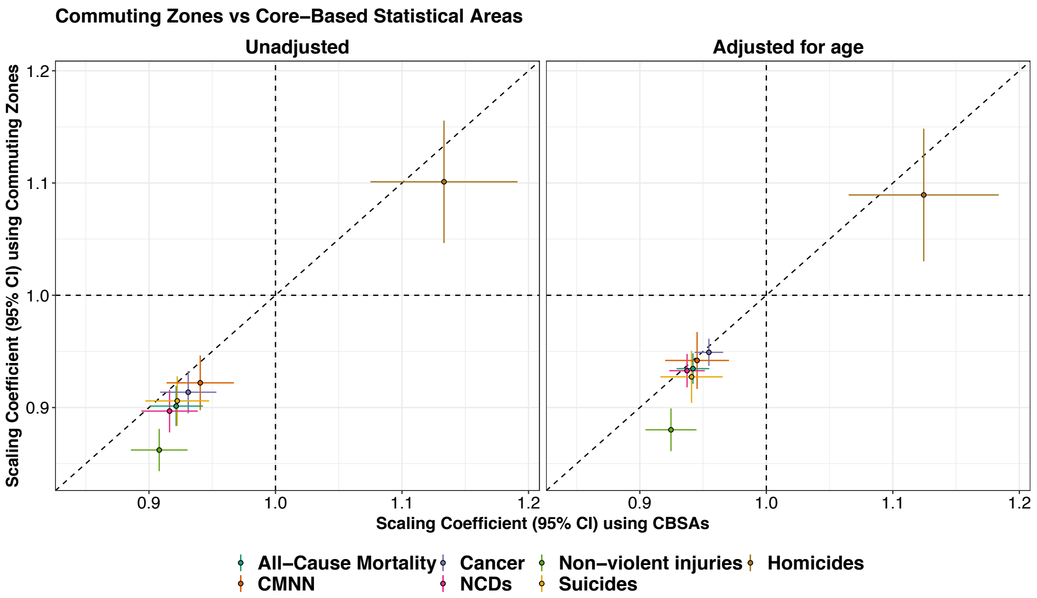

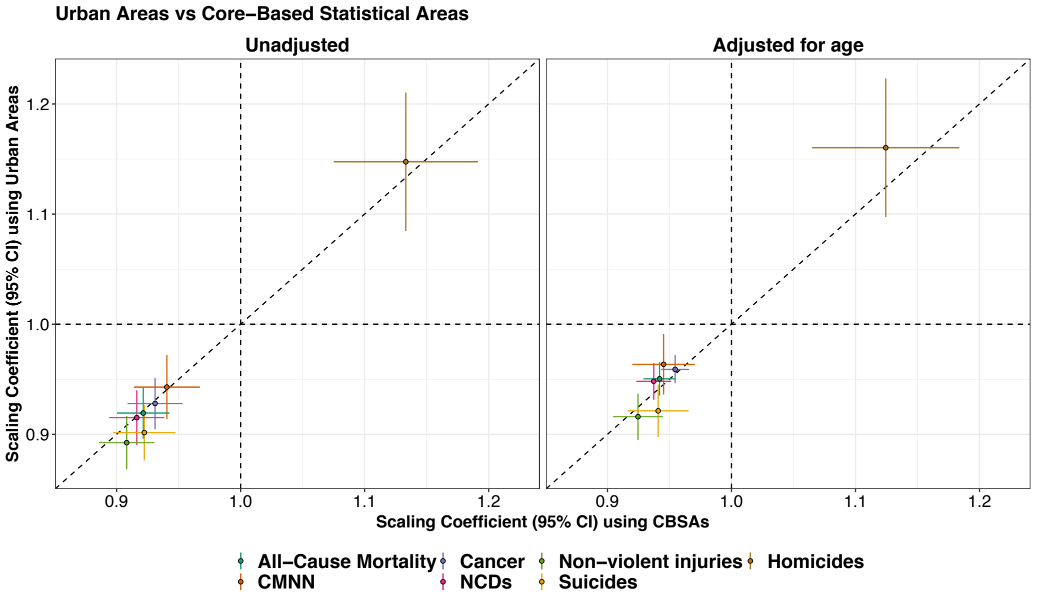

Supplementary Figure S3: comparison of scaling coefficients in the main models and after adjusting for a covariate indicating whether each city is the largest city in its country

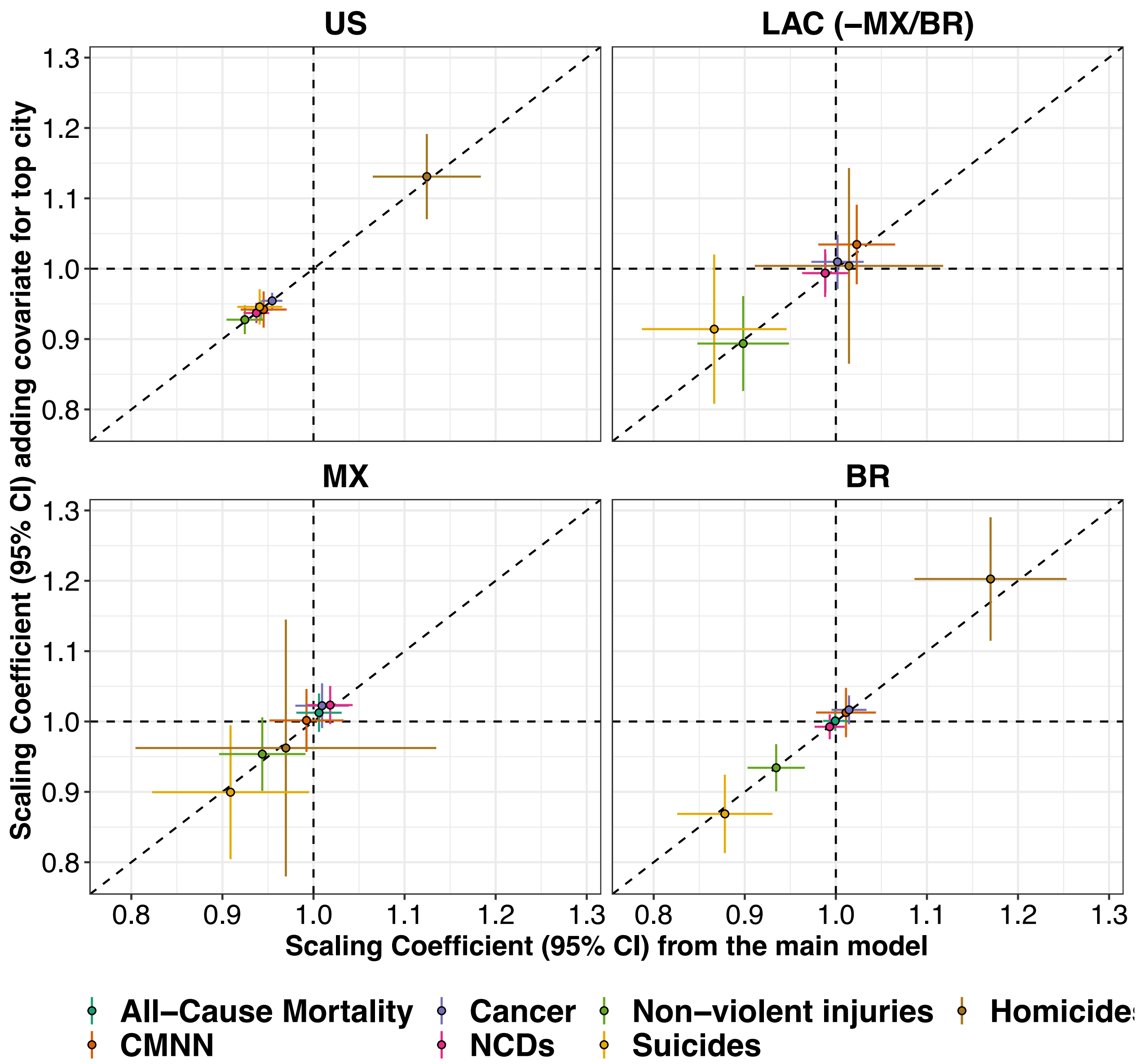

Supplementary Table S6: City Definitions used in this study

Area	Name	Definition	Source
Latin America	SALURBAL City	Aggregation of county-like units (*municipios*, *comunas*, etc.) that overlap with the urban footprint of each city	SALURBAL Study Protocol (71)
US (main analysis)	CBSA	Aggregation of counties around a core area above 50,000 residents, with high commuting interchange	Sept 2018 CBSA delineations (https://www.census.gov/geographies/reference-files/time-series/demo/metro-micro/delineation-files.html)
US (sensitivity analysis)	Commuting Zone	Aggregation of counties with high commuting interchange	2010 version by Fowler et al (77) (available at: https://sites.psu.edu/psucz/)
US (sensitivity analysis)	Urban Area (proxy)	Aggregation of counties that overlap census-defined Urban Areas	2018 version of Cartographic Boundary Files (https://www.census.gov/geographies/mapping-files/time-series/geo/carto-boundary-file.html)

Footnote: In all cases, we restricted the analysis to cities (in each definition) with >100,000 residents.
